# Early immune response to *Toxoplasma gondii* lineage III isolates of different virulence phenotype

**DOI:** 10.3389/fcimb.2024.1414067

**Published:** 2024-06-07

**Authors:** Aleksandra Uzelac, Ivana Klun, Olgica Djurković-Djaković

**Affiliations:** Institute for Medical Research, University of Belgrade, Belgrade, Serbia

**Keywords:** *Toxoplasma gondii*, immune response, cytokines, virulence, genotypes

## Abstract

**Introduction:**

*Toxoplasma gondii* is an intracellular parasite of importance to human and veterinary health. The structure and diversity of the genotype population of *T. gondii* varies considerably with respect to geography, but three lineages, type I, II and III, are distributed globally. Lineage III genotypes are the least well characterized in terms of biology, host immunity and virulence. Once a host is infected with *T.gondii*, innate immune mechanisms are engaged to reduce the parasite burden in tissues and create a pro-inflammatory environment in which the T_H_1 response develops to ensure survival. This study investigated the early cellular immune response of Swiss-Webster mice post intraperitoneal infection with 10 tachyzoites of four distinct non-clonal genotypes of lineage III and a local isolate of ToxoDB#1. The virulence phenotype, cumulative mortality (CM) and allele profiles of ROP5, ROP16, ROP18 and GRA15 were published previously.

**Methods:**

Parasite dissemination in different tissues was analyzed by real-time PCR and relative expression levels of IFNγ, IL12-p40, IL-10 and TBX21 in the cervical lymph nodes (CLN), brain and spleen were calculated using the ΔΔCt method. Stage conversion was determined by detection of the BAG1 transcript in the brain.

**Results:**

Tissue dissemination depends on the virulence phenotype but not CM, while the TBX21 and cytokine levels and kinetics correlate better with CM than virulence phenotype. The earliest detection of BAG1 was seven days post infection. Only infection with the genotype of high CM (69.4%) was associated with high T-bet levels in the CLN 24 h and high systemic IFNγ expression which was sustained over the first week, while infection with genotypes of lower CM (38.8%, 10.7% and 6.8%) is characterized by down-regulation and/or low systemic levels of IFNγ. The response intensity, as assessed by cytokine levels, to the genotype of high CM wanes over time, while it increases gradually to genotypes of lower CM.

**Discussion:**

The results point to the conclusion that the immune response is not correlated with the virulence phenotype and/or allele profile, but an early onset, intense pro-inflammatory response is characteristic of genotypes with high CM. Additionally, high IFNγ level in the brain may hamper stage conversion.

## 1 Introduction

*Toxoplasma gondii* is a generalist zoonotic protozoan parasite with a complex life cycle involving a large number of mammal and bird species as intermediate hosts and only the Felidae as definitive hosts. *T. gondii* is transmitted by food and water and is capable of surviving in the environment under various conditions. Several hundred genotypes of *T. gondii* have been identified and population genetics revealed a structure of six clades made up of a number of lineages and fifteen defined haplogroups (Shwab et al., 2014). Of the genotypes, only three are known as the archetypes, the clonal genotypes ToxoDB#10 (type I), ToxoDB#1 (type II) and ToxoDB#2 (type III), which represent the three globally distributed lineages. The archetypes differ by virulence phenotype and frequency of occurrence. As they were among the first to be isolated and cultivated in the laboratory, they have become the model parasites. Natural infection with *T. gondii* primarily occurs through ingestion of food and/or water in which tissue cysts which contain dormant bradyzoites or oocysts which contain sporozoites, a developmental stage which occurs after sexual reproduction of the parasite in definitive hosts, are present. Although less frequent, vertical transmission of the free-living stage (tachyzoites) from a gravid host to the offspring is also possible (Tenter et al., 2000; Hill and Dubey, 2002). Once the host becomes infected, the immune system is permanently activated, since the parasites are never eliminated, partly due to intracellular encystation preferentially in neurons (Ferguson and Hutchison, 1987; Fischer et al., 1997; Cabral et al., 2016). Generally, immunocompetent individuals may be asymptomatic or have only mild symptoms during acute infection and remain without clinical manifestation for life without any medical treatment. Direct interactions between the parasite and host immune cells occur primarily during early infection, during the parasite’s tachyzoite stage. As tachyzoites invade and kill infected cells and/or are digested, parasite proteins are secreted along with immunostimulatory molecules and various biological debris which act as antigen and/or ligands. Upon stage conversion into bradyzoites, the repertoire of available proteins changes. Remarkably, this occurs apparently within three to five days post inoculation, as evident through observation of tissue cysts in the brain of experimentally infected laboratory mice (Dubey et al., 1998). Along with responses in the brain, those mediated by cervical lymph nodes (CLN) and the spleen are important in the context of early *T. gondii* infection (Glatman Zaretsky et al., 2012; Lee et al., 2019; Pereira et al., 2019; Kovacs et al., 2022). Experimental infection models with archetypal strains using laboratory mice showed that the immune response varies by parasite genotype, while survival is determined by its virulence phenotype (Sibley and Boothroyd, 1992; Zhang et al., 2018). Most mouse strains have a 1% chance of survival when infected by acutely virulent genotypes such as ToxoDB#10 and up to 70% with genotypes of low virulence, such as ToxoDB#2, while survival chances after infection with intermediately virulent genotypes (ToxoDB#1) are somewhere in between (Sibley and Boothroyd, 1992). Immunity is mediated through the development of the CD4+ T helper cell type 1 response (T_H_1), which depends on early secretion of pro inflammatory cytokines, most importantly IFNγ by group 1 innate lymphoid (ILC1) cells, which is critical for host survival (Suzuki et al., 1988; Denkers et al., 2004; Goldszmid et al., 2012; López-Yglesias et al., 2018). IFNγ production is induced by IL-12, secreted primarily by dendritic cells (DC), specifically cDC1 in mice (Sher et al., 2017). As overproduction of IFNγ and other pro inflammatory cytokines can lead to immunopathology, the intensity of the T_H_1 response must be efficiently controlled (Yap and Sher, 1999; Sasai and Yamamoto, 2019; Khan and Moretto, 2022). A key cytokine which dampens the pro inflammatory response in *T. gondii* infection is IL-10, secreted early on by macrophages and later by lymphocytes. Macrophage interaction with *T. gondii* tachyzoites, immune responses and activation phenotype (classical, M1 or alternative, M2), have been shown to be related to the virulence of the infecting genotype (Jensen et al., 2011; Zhao et al., 2014; Hassan et al., 2015). The transcription factor TBX21, or T-bet, is a lineage-determining factor for multiple immune cell types (CD4, CD8, NKT, NK and B cells), induced by inflammatory signals to coordinate different transcriptional programs for effective innate and adaptive immunity to intracellular pathogens (Kallies and Good-Jacobson, 2017; Pritchard et al., 2019; Hertweck et al., 2022). Although the significance of T-bet in facilitating polarization of CD4+ to the T_H_1 phenotype through activation of IFNγ and other pro inflammatory cytokines was established long ago (Lighvani et al., 2001; Denkers et al., 2004), it has been recently demonstrated to be critical for the maintenance of DCs during early *T. gondii* infection (Mashayekhi et al., 2011; López-Yglesias et al., 2018). Early cellular immune response to *T. gondii* tachyzoites is initiated in part through the effects exerted by intra-and extracellularly secreted parasite ‘effectors’, rhoptry proteins (ROP) and dense granules (GRA), some of which have been identified as virulence factors, such as ROP5, ROP16, ROP18 and GRA12 and GRA15 (Su et al., 2002; Saeij et al., 2006; Young et al., 2019; Lockyer et al., 2023). Some, like GRA12, appear to be ‘universal’ virulence factors, mediating virulence independently of the murine host genetics, while others manifest virulence only in a specific genetic context. In general, virulence factors promote parasite survival through blocking, activating and/or interfering with host cell protein binding, signaling and/or transcription. Sequence and functional analyses of these virulence factors in the archetypes revealed the existence of different alleles (I, II, III) associated with specific host immune responses. For instance, ROP18 I and II phosphorylate IRG proteins and prevent GTPase function, thus protecting the parasitophorous vacuole from destruction, while ROP18 III is not expressed. The activity of ROP18 is modulated by the pseudokinase ROP5, with archetypes I (ToxoDB#10) and III (ToxoDB#2) having similar ROP5 allele clusters, different from archetype II (ToxoDB#1), but each archetype has a different locus structure (Reese et al., 2011; Behnke et al., 2012). ROP16 I and III, but not ROP16 II, constitutively activate STAT3/6 in immune cells, thereby decreasing IL-12 production by macrophages, inducing arginase (ARG1) and shutting down nitric oxide synthase (NOS2) production. This leads to the polarization of macrophages to the alternatively activated (M2) phenotype, associated with wound healing and tolerance instead of microbicidal activity (Butcher et al., 2011; Jensen et al., 2011). GRA15 II, but not the other two alleles, induces NF-κB translocation, which results in the production of pro- and anti-inflammatory cytokines, including IL-12. While the archetypes have been instrumental in elucidating cellular immunity to *T. gondii*, a far greater diversity of mechanisms and responses are to be expected with non-clonal genotypes. As a number of non-clonal genotypes are virulent in mice, and natural infection with non-clonal genotypes is more common than previously assumed, understanding immunity to non-clonal genotypes is important (Marković et al., 2014; Pomares et al., 2018; Hamilton et al., 2019; Pardini et al., 2019; Schumacher et al., 2021; Castillo-Cuenca et al., 2023). In this study, the early immune response to infection with four distinct non-clonal lineage III genotypes of different virulence phenotypes was investigated through the relative expression of IFNγ, IL12-p40, IL-10 and T-bet in the CLN, brain and spleen of Swiss Webster (SW) mice, along with parasite tissue distribution and stage conversion.

## 2 Materials and methods

### 2.1 Mice

SW female mice weighing between 20 - 25g were purchased from the Military Medical Academy in Belgrade and transferred to the Animal Research Facility at the Institute for Medical Research (IMR). The mice were acclimated in communal cages (10 per cage) for 7 – 10 days prior to infection with *T. gondii* tachyzoites and subsequently transferred to smaller cages (5 per cage) for the duration of the experimental infection period. Mice were given water and food (pellets) ad libitum and were kept at a natural daylight cycle. The cages were cleaned and fresh bedding, water and food were provided twice per week. Animals with injuries and/or those which displayed aggressive behavior were excluded prior to infection, while the onset and severity of any clinical symptoms was monitored and assessed daily during the infection period. Animal experiments were approved by the Ethics Council of the Ministry of Agriculture, Forestry and Water Management of Serbia Veterinary Directorate (Decision no. 323–07-05567/2019–05 of 10 July 2019).

### 2.2 Parasite strains

The genotypes used in this study were all previously described (Uzelac et al., 2020). *T. gondii* genotypes G13 (Marković et al., 2014), EQ39 (ToxoDB#54), EQ 40 (Klun et al., 2017) and K1 were isolated from animal hearts and are of lineage III. BGD18 (ToxoDB#1) was isolated from a patient (Uzelac et al., 2020). The isolation protocol is described in Djurković-Djaković et al. (2005). All isolates except G13 were maintained *in vivo* for a brief period after isolation by serial, oral passage of tissue cysts in SW mice (five passages at an interval of 6 months) before conversion into tachyzoites and freezing for long term storage. G13 was passaged 15 times. Each isolate was briefly propagated in VERO cells *in vitro* (4–6 days), to obtain tachyzoites suitable for experimental infections described herein. The conversion protocol from tissue cysts (bradyzoites) to tachyzoites and *in vitro* propagation are described in detail in Uzelac et al. (2020). The CM of SW mice induced by intermediately virulent genotypes EQ40 and K1 were 69.4% and 38.8%, with (LD_50_) of 10^2^ and 10^4^, respectively (Uzelac et al., 2020). The CM of the low virulence genotypes G13 and EQ39 (ToxoDB#54) were 10.7% and 6.8%, respectively. The low virulence phenotype of isolate BGD18 (ToxoDB#1) was experimentally confirmed. Proliferation rate, lytic capacity as well as ENO2 expression were shown to be higher in isolates of intermediate virulence (Uzelac et al., 2020).

### 2.3 Infection protocol

Infections were performed by intraperitoneal inoculation of 500 µL of a suspension containing 10 tachyzoites of each isolate and gentamicin (1.6 mg/kg) in sterile saline (Hemofarm, Vršac, Serbia) into each mouse (n=17 per isolate) using an insulin syringe and 18 G needle.

### 2.4 Organ extraction and homogenization

Per each time point (24h, 3d, 7d and 10 d), 3–5 SW mice infected with each isolate were sacrificed by cervical dislocation. Cervical lymph nodes (CLN), the thymus along with mediastinal lymph nodes (ThyMLN), brains (B) and spleens (S) were extracted using sterile surgical instruments. The organs were rinsed with saline (Hemofarm, Vršac, Serbia) and transferred into 2 ml screw-cap tubes containing sterile 1.4 mm ceramic beads (Omni International, Kennesaw, GA, USA) and 1 ml of Trizol reagent (Invitrogen, Carlsbad, CA). Mechanical homogenization was performed in a Bead Ruptor instrument (Omni International, Kennesaw, GA, USA) using the maximum setting for 30 sec. The homogenates were frozen at -20°C for short term, or -70°C for long term storage until nucleic acid extraction.

### 2.5 Extraction of nucleic acids and cDNA synthesis

The extraction of gDNA and total mRNA from whole organ suspensions using the Trizol reagent were performed according to the manufacturer’s instructions. The gDNA was resuspended in 300 µL of 0.8 mM NaOH solution, while the mRNA pellet was resuspended in 200 µL of nuclease free water and the concentration was estimated using the Qubit 2.0 fluorimeter (Invitrogen, Carlsbad, CA) according to the manufacturer’s instructions. Extractions were run in batches of 23 samples, while one extraction control was included in each run (organ homogenate from uninfected mouse used as calibrator). 0.5–2 µg of total RNA was used for conversion into first strand cDNA using the Revert Aid First Strand cDNA synthesis kit (Thermo Fisher Scientific, Waltham, MA, USA) according to the manufacturer’s instructions. To capture mRNA, the random hexamer oligo was replaced with OligoDT (Thermo Fisher Scientific, Waltham, MA, USA).

### 2.6 Detection of parasite gDNA

Detection of *T. gondii* gDNA in organ homogenates targeted the 529bp repetitive element (RE) (AF146527). Each PCR reaction consisted of 10 μL TaqMan Universal PCR Mastermix (Applied Biosystems, Foster City, CA, USA), 0.25 mM forward (F) and reverse (R) primers 5′-AGA GAC ACC GGA ATG CGA TCT-3′; 3’-CCC TCT TCT CCA CTC TTC AAT TCT-5′), 0.10 mM of the specific TaqMan probe FAM-ACG CTT TCC TCG TGG TGA TGG CG-TAMRA (Invitrogen, Life Technologies, Carlsbad, CA, USA) and 3 μl of extracted gDNA (Lélu et al., 2011). An exogenous internal reaction control was added to each reaction (Thermo Fisher Scientific, Waltham, MA, USA). The final reaction volume was 20 μl. The thermal cycling program consisted of the following steps: 5 min at 95 °C for initial denaturation, followed by 45 cycles of 15 s at 95 °C for denaturation and 60 s at 60 °C for annealing/extension. Detection occurred at the end of the 60 °C annealing/extension step. Amplification and detection were performed in a StepOnePlus Real Time PCR System (Applied Biosystems, Foster City, CA, USA). Each sample was run in triplicate and evaluated as +/- (presence/absence) of *T. gondii* gDNA. The final result represents the decision call when 2/3 results matched. Samples were run in batches on plates, with each plate also containing several positive (*T. gondii* gDNA isolated from pure tachyzoites) and negative control reactions (nuclease-free water).

### 2.7 Relative quantitation of gene expression

Expression primers were designed using published sequences (**Table 1**), annealing temperatures were adjusted to 55°C. The relative expression levels of murine IL-10, IL-12p40 and IFN-γ were normalized to β-actin while cDNA synthesized from organs of uninfected SW mice (n=5) was used for calibration. Relative quantity was calculated based on the ΔΔCt method with kinetic PCR efficiency correction. Each PCR reaction contained 10 µl of PowerUp SYBR Green Mastermix (Applied Biosystems, Foster City, CA, USA), 20 pmol of each F and R primer (sequences are shown in table below) 2 µl of cDNA template and RNAse and DNAse free water in a final volume of 20 µl. The thermal profile consisted of an initial holding step, 2 min at 50°C to activate UDG, followed by polymerase activation for 2 min at 95°C and 40 cycles of three step PCR: 15 sec at 95°C, 15 sec at 55°C, 60 sec at 72°C. Data collection was performed during the extension step. Melting curves were generated after each run using a default thermal dissociation profile in the StepOnePlus instrument (Applied Biosystems, Foster City, CA, USA) software. Each reaction was run in triplicate. SAG1 and BAG1 mRNA were amplified using the same protocol, but relative expression was not evaluated, instead data were evaluated as presence or absence of transcript. The positive control for SAG1 expression were tachyzoites of the RH strain (ToxoDB#10), while the positive control for BAG1 expression were tissue cysts of the Me49 strain purified from chronically infected SW mice. Melting curves were analyzed to verify the presence of specific peaks (≥80°C) for the analyzed transcripts.

**Table 1:** Primer sequences used in this study.

Target	Forward primer (3`→ 5`)	Reverse primer (3`→ 5`)
β-actin	CACCACAGCTGAGAGGGAAATC	GTTTCATGGATGCCACAGGATTCC
IL-10	CTGTCATCGATTTCTCCCCTGTG	GACTCAATACACACTGCAGGTG
IL-12p40	GTATTCAGTGTCCTGCCAGGAG	GTCTGGTTTGATGATGTCCCTG
IFN-γ	GAAAGACAATCAGGCCATCAGC	CGGATGAGCTCATTGAATGC
TBX21 (T-bet)	CAACAACCCCTTTGCCAAAG	GGAACTCCGCTTCATAACTGTG
TgBAG1	GGAGCCATCGTTATCAAAGGAG	GATTCCGTCGGGCTTGTAATTAC
TgSAG1	CTTGCGATGTGGCGTTAT	GCTTCAGGAATCAAGGAGCTC

### 2.8 Statistical analyses

Statistical analyses and graphical representation of the relative expression data were performed using GraphPad Prism 8. Error bars represent the standard error of the mean (SEM) derived from analyzing 3–5 mice. The expression was analyzed using one-way ANOVA and the Bonferroni post test was applied. The differences were considered significant when p < 0.05 (*).

## 3 Results

### 3.1 Dissemination kinetics

*T. gondii* gDNA was detected in the least number of tissue samples (39%) 24 h post- infection (p.i.) and in the greatest number of samples on day 7 (65%) and day 10 (64%) (**Table 2**). Differences between genotypes of low virulence, BGD18 (ToxoDB#1), G13 and EQ39 (ToxoDB#54) and intermediate virulence, K1 and EQ40, were detected at all time points after infection. At the earliest time point, parasite gDNA was detected in 30% versus 50% of tissues of animals infected with genotypes of low and intermediate virulence, respectively. At 3 days and 7 days after infection, *T. gondii* gDNA was detected in a greater number of tissues (61% and 71% respectively) of animals infected with genotypes of low virulence as opposed to animals infected with intermediately virulent ones (50% and 52% respectively). At the final time point, *T. gondii* gDNA was present in a greater number of tissues in animals infected with intermediately virulent genotypes (68% versus 46%). The tissues of two animals infected with BGD18 were found to be free of parasites 24 h (mouse 2) and 3 days (mouse 1) p.i., however, as only four tissues were examined, these animals were not omitted from the data analysis. Tissues of one animal (mouse 3 infected with EQ40) were found to be free of parasite gDNA 10 days post infection, and as this is a late time point, at which tissue cysts should be present in the brain, this most likely indicates that it may not have been infected, and it was therefore excluded from the data analysis and interpretation.

**Table 2:** Distribution kinetics of *T. gondii* 24h, 3d, 7d and 10d post infection in cervical lymph nodes (CLN), thymus and mediastinal lymph nodes (Thy/MLN), spleen (S) and brain (B).

Time point	Tissue	*T. gondii* genotypes														
		BGD18 (ToxoDB#1)			G13			EQ39 (ToxoDB#54)			K1			EQ40		
24 h	CLN	+	†	+	–	–	+	–	–	+	+	+	+	+	+	–
	Thy/MLN	–	†	–	–	+	–	–	+	–	+	–	+	–	–	–
	S	+	†	–	–	–	–	+	na	–	+	+	–	+	–	–
	B	–	†	–	+	–	–	–	+	–	–	–	+	+	–	+
3 d	CLN	†	–	–	–	–	+	+	+	–	+	+	–	+	+	+
	Thy/MLN	†	–	+	+	+	na	+	–	–	+	–	–	–	–	–
	S	†	+	–	+	+	–	–	–	–	+	–	–	–	–	–
	B	†	+	+	+	+	+	+	+	+	+	+	+	+	+	–
7 d	CLN	+	–	+	+	+	+	–	+	–	+	+	+	+	+	+
	Thy/MLN	+	–	na	–	–	–	–	–	+	–	–	–	–	na	–
	S	+	+	na	+	+	+	+	+	+	–	–	+	+	+	+
	B	+	+	+	+	+	+	+	+	+	–	–	–	+	–	+
10 d	CLN	+	+	+	–	+	–	+	+	–	+	na	+	+	+	†
	Thy/MLN	+	+	+	–	–	–	–	–	–	+	+	–	–	–	†
	S	+	+	+	–	+	+	–	–	–	+	+	–	+	–	†
	B	+	+	+	+	+	+	+	+	+	+	–	+	+	+	†

Each column represents one individual mouse, + represents tissues in which *T. gondii* gDNA was detected and - represents no detection. No amplification (designated as na in the table) of the internal control was considered a failed reaction. † indicates gDNA was not detected in any of the investigated tissues.

### 3.2 Stage conversion

Detection of BAG1 transcripts in brain homogenates was done at all time points p.i., however the first time it was detected in any of the mice was at day 7. Detection was overall more successful 10 days p.i. than 7 days p.i. (**Table 3**). The expression of SAG1, which was performed as a control, was not detected before day 7, then sporadically 7 days p.i. and more consistently 10 days p.i. (**Table 2**). BAG1 was successfully detected in 2/3 the brain homogenates of mice infected with BGD18 (ToxoDB#1) and in 1/3 infected with EQ39 (ToxoDB#54) 7 days p.i. At 10 days p.i., BAG1 was detected in all three mice infected with BGD18 (ToxoDB#1) and 2/3 mice infected with K1.

**Table 3:** SAG1 and BAG1 mRNA presence in brain homogenates in which *T. gondii* gDNA was detected, 7d and 10d post infection.

	*T. gondii genotypes*														
	BGD18 (ToxoDB#1)			G13			EQ39 (ToxoDB#54)			K1			EQ40		
7d	**SAG+**BAG-	SAG-**BAG+**	SAG-**BAG+**	SAG-BAG-	SAG-BAG-	SAG-BAG-	SAG-BAG-	**SAG+BAG+**	SAG-BAG-	–	–	–	SAG-BAG-	–	SAG-BAG-
10d	**SAG+BAG+**	**SAG+BAG+**	**SAG+BAG+**	**SAG+**BAG-	**SAG+**BAG-	SAG-BAG-	SAG-BAG-	SAG-BAG-	SAG-BAG-	**SAG+BAG+**	–	**SAG+BAG+**	SAG-BAG-	SAG-BAG-	–

### 3.3 Kinetics of cytokine expression

The results of the experiments described in sections (b) and (c) are based on the dissemination results (**Table 2**).

#### 3.3.1 In cervical lymph nodes

**Figure 1:**
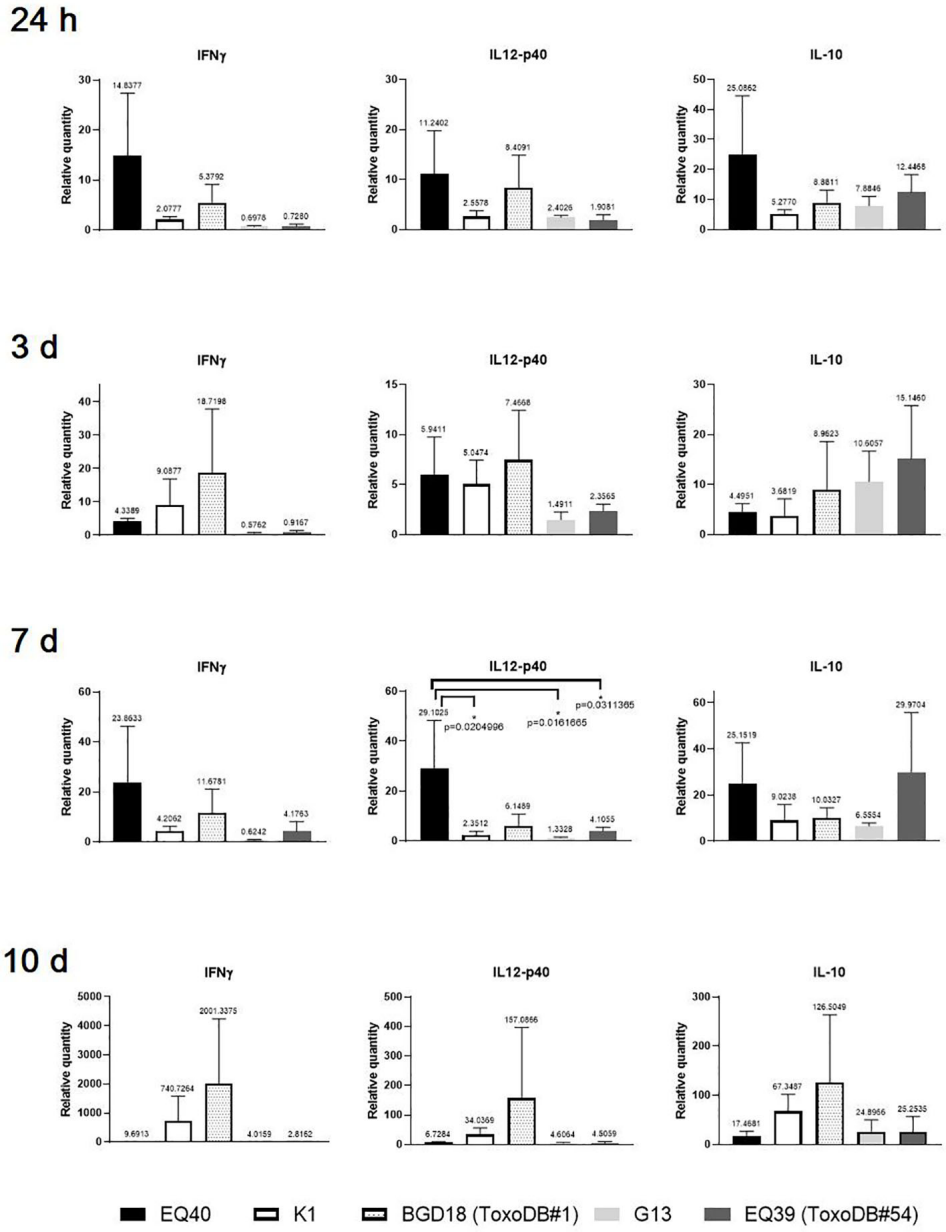
Relative expression levels of IFNγ, IL12-p40 and IL-10 in CLN 24 h and 3, 7 and 10 days post infection. Bars from left to right correspond to: EQ40, K1, BGD18 (ToxoDB#1), G13, EQ39 (ToxoDB#54). The error bars represent SEM, results were analyzed by one-way ANOVA with Bonferroni *post-hoc* test (*p < 0.05). P values are shown on the respective graphs, fold change is indicated above bars.

At the earliest time point (24 h) p.i., an up-regulation of the expression levels of IL12-p40 and IL-10 was detected in the CLN of the majority of the mice (**Figure 1**), while IFNγ was up-regulated only in the mice infected with EQ40, K1 and BGD18 (ToxoDB#1) and down-regulated in the mice infected with G13 and EQ39 (ToxoDB#54). While the levels varied considerably, none of the differences in cytokine expression were statistically significant at 24 h and 3 days p.i. At 24 h p.i. the highest level of IFNγ was detected in mice infected with EQ40, while the highest level of IL12-p40 was detected in the mice infected with BGD18 (ToxoDB#1) and of IL-10 in mice infected with EQ39 (ToxoDB#54). Three days p.i., the expression of all three cytokines in the CLN of mice infected with EQ40 decreased as compared to the 24 h time point, most notably of IL-10. IFNγ was at this time point still down-regulated in the mice infected with G13 and EQ39 (ToxoDB#54) while IL-10 increased as compared to the 24 h time point. The differences in expression of IL12-p40 between mice infected with different genotypes reached statistical significance only 7 days p.i. The highest level was present in the mice infected with EQ40. At the same time point, the level of IFNγ was up-regulated for the first time in the CLN of mice infected with EQ39 (ToxoDB#54), while the level of IFNγ was still down-regulated in the mice infected with G13. There was an increase in the level of IL-10 in the CLN of all mice except for those infected with G13 as compared to the previous time point, with the highest level present in the mice infected with EQ39 (ToxoDB#54). At the final time point, day 10 p.i., there was a marked increase in expression of all three cytokines in the mice infected with BGD18 (ToxoDB#1) and K1, while a notable increase in the level of IL-10 was detected in the CLN of mice infected with G13, although the differences were not statistically significant due to considerable heterogeneity among the mice.

#### 3.3.2 In brain and spleen 7 days post infection

**Figure 2:**
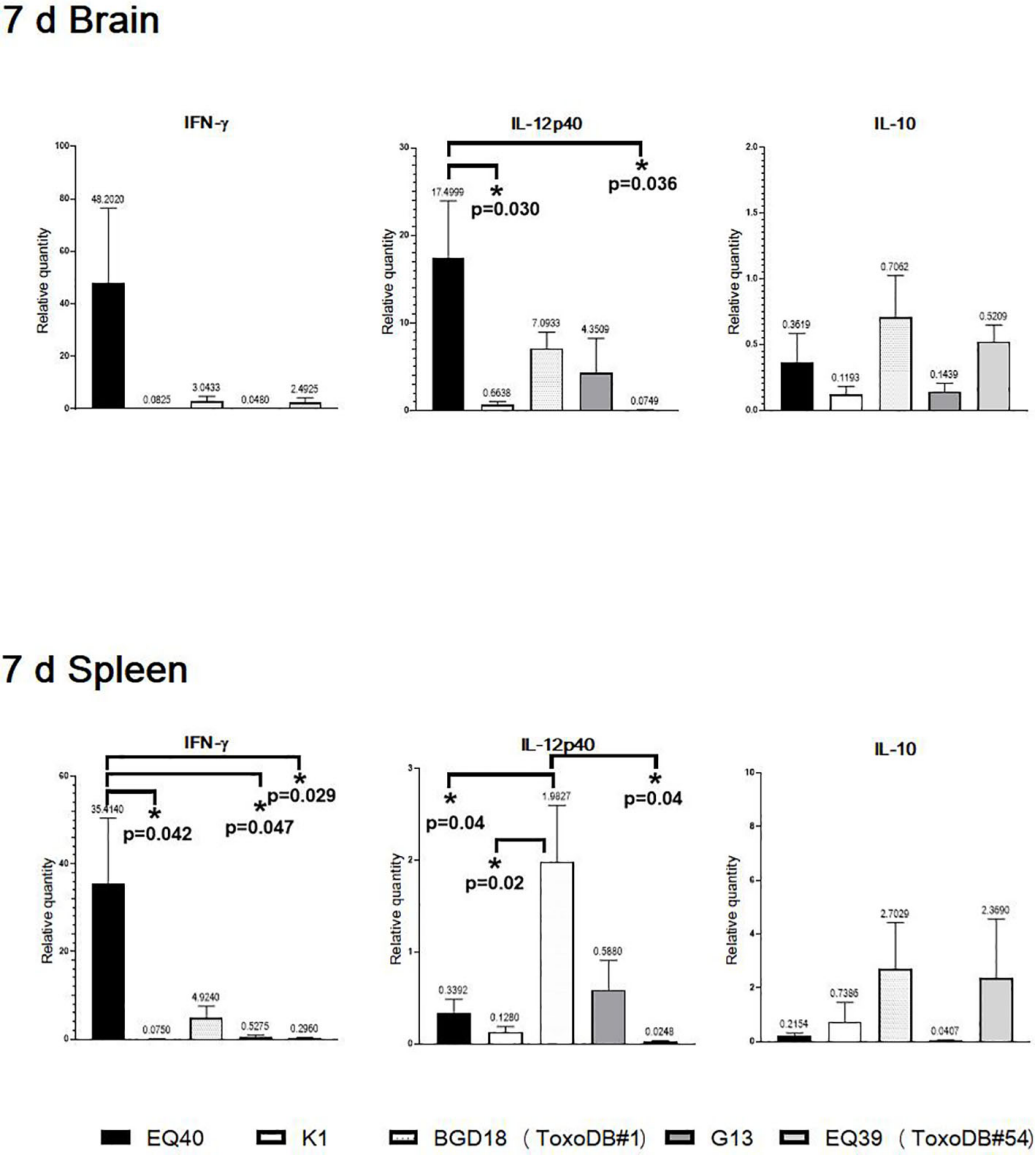
Relative expression levels of IL12-p40, IFNγ and IL10 in brain and spleen 7 days post infection. Bars from left to right correspond to: EQ40, K1, BGD18 (ToxoDB#1), G13, EQ39 (ToxoDB#54). The error bars represent SEM, results were analyzed by one-way ANOVA with Bonferroni *post-hoc* test (*p < 0.05). P values are shown on the respective graphs, fold change is indicated above bars.

The expression at the 7 day time point p.i. was selected based on the dissemination results. The highest level of IFN was detected in brain tissues and spleens of the mice infected with EQ40, but reached statistical significance only in the spleens (**Figure 2**). IFN-γ was down-regulated in the brain tissues and spleens of the mice infected with K1 and G13, and also in the spleens of the mice infected with EQ39 (ToxoDB#54). The IL12-p40 level was statistically significantly higher in the brain tissues of the mice infected with EQ40, and down-regulated with K1 and EQ39 (ToxoDB#54), while IL-10 was down-regulated by all genotypes. IL12-p40 was statistically significantly higher only in the spleens of the mice infected with BGD18 (ToxoDB#1), but the level was much lower than in the brain tissues. IL-10 was down-regulated in the brain tissues of the mice infected with EQ40, K1 and G13 while low levels were detected in the brain tissues of the mice infected with BGD18 (ToxoDB#1) and EQ39 (ToxoDB#54).

#### 3.3.3 Kinetics of T-bet expression in cervical lymph nodes

**Figure 3:**
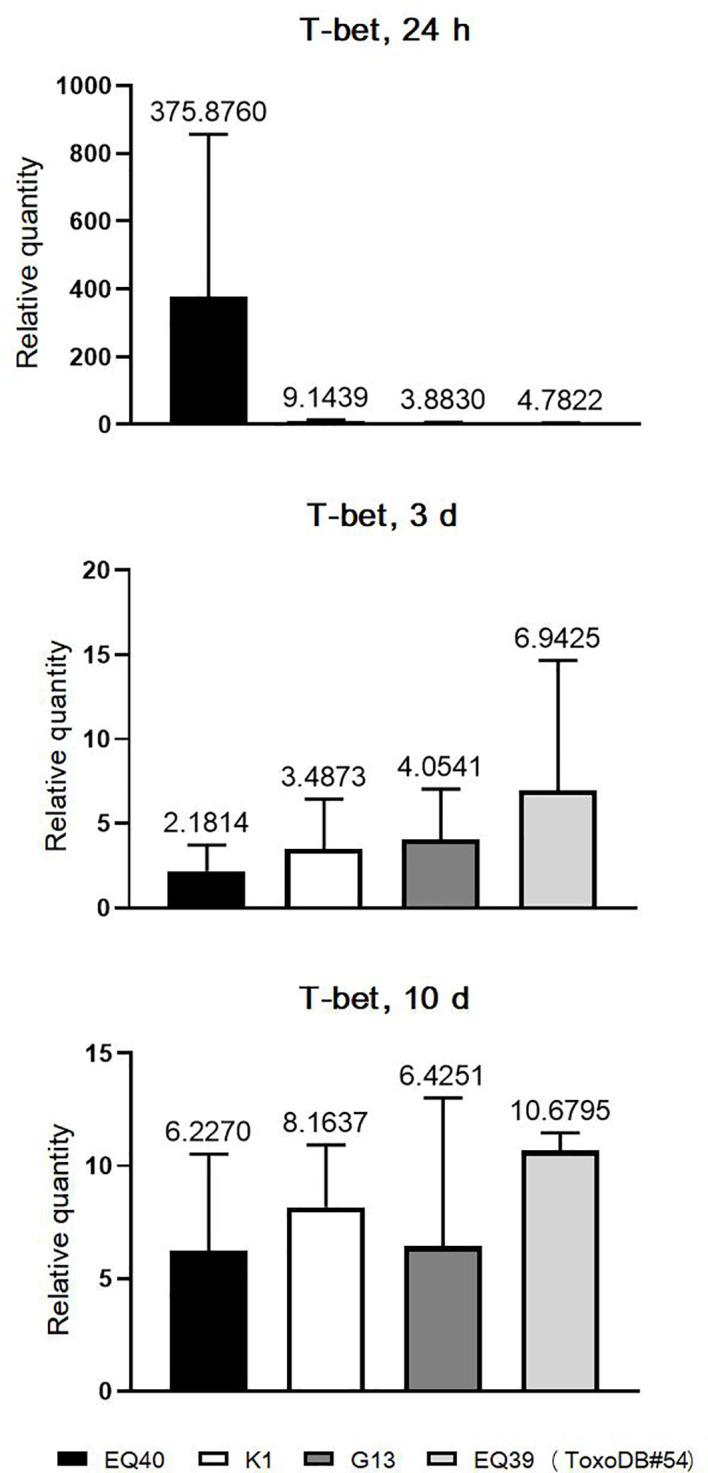
Relative expression levels of T-bet in CLN 3 and 10 days p.i. Bars from left to right correspond to: EQ40, K1, BGD18 (ToxoDB#1), G13, EQ39 (ToxoDB#54). The error bars represent SEM, results were analyzed by one-way ANOVA with Bonferroni *post-hoc* test. Fold change is indicated above bars.

The kinetics of T-bet expression were analyzed only in the CLN, due to the very early (24 h p.i.) presence of tachyzoites in this tissue in most of the infected mice. T-bet was analyzed only in the mice infected with the lineage III genotypes (**Figure 3**). The results indicate that T-bet is up-regulated immediately after infection and remains up-regulated until 10 days p.i. At the 24 h time point, the highest level of T-bet expression was detected in the mice infected with EQ40. Overall, the levels were higher in both intermediately virulent genotypes as compared to the genotypes of low virulence. At three days p.i., the levels of T-bet in the CLN of mice infected with the genotypes of low virulence had increased, while there was a decrease in the CLN of mice infected with the intermediately virulent genotypes. At the final time point, 10 days p.i., the levels were similar.

## 4 Discussion

In this study, the early immune response elicited by four non-clonal genotypes of *T. gondii* lineage III was investigated through the relative expression of key cytokines (IFNγ, IL12-p40 and IL-10) and the transcription factor, T-bet. Dissemination of tachyzoites was analyzed by detection of parasite gDNA in different tissues, while the presence of BAG1 transcript, the canonical marker for tachyzoite to bradyzoite conversion, was analyzed in the brain as the preferred site of cyst formation/localization. As the genotypes used herein all have identical alleles of ROP5 (III), ROP18 (III), ROP16 (I/III) and GRA15 (I), as reported earlier, a low passage isolate of ToxoDB#1 (BGD18) with allele II at all loci, was used for comparison (Uzelac et al., 2020). We here used a low dose infection model (10 tachyzoites/mouse), which is not common, but was necessary due to the low LD_50_ of EQ40 (10^2^) in order to avoid overt clinical symptoms and high inflammation, especially as intraperitoneal infection has been shown to mediate a more intense inflammatory response than oral infection (French et al., 2022). Lymphadenomegaly and/or splenomegaly, which are common even at early time points in infected mice, were not observed consistently at any time point after infection, nor could a correlation be made with the *T. gondii* genotype and/or virulence phenotype. This is in contrast with the results of Zhang et al. (2018), who observed consistent splenomegaly seven and nine days p.i. with RH (ToxoDB#10) and Me49 (ToxoDB#1). The most likely explanation is the difference in the inoculum size, as Zhang et al. (2018) used 100 parasites versus the 10 parasites used in this study.

Predictably, there was a marked increase in our ability to detect parasite DNA as the infection progressed, due to proliferation and accumulation of tachyzoites in host tissues over time (**Table 2**). When analyzing the numbers of samples with detectable parasite DNA by time point, the results indicate that dissemination of tachyzoites of intermediately virulent genotypes, EQ40 and K1, peaks very early (24 h) p.i. and remains fairly constant throughout the observed time period, whereas dissemination of tachyzoites of low virulence shows an increasing trend, peaking seven days p.i. and decreasing thereafter. Interestingly, the tissues in which parasite gDNA was detected most frequently 24 h p.i. were the CLN, three days p.i. it was the brain, while seven and ten days p.i., the CLN and the brain had equalized. Parasite gDNA was consistently detected in the spleens of the majority of infected animals only at the later time points, seven and ten days p.i., while the detection in the Thy/MLN was sporadic at all time points. Although different genotypes, infection models and experimental approaches have been used here and in Chiebao et al. (2021), the combined results indicate that more virulent non-archetypal genotypes may disseminate more rapidly as compared to those of low virulence, which has been postulated by a number of earlier studies using archetypes (Barragan and Sibley, 2002; Saeij et al., 2005; Djurković-Djaković et al., 2012). Differences in dissemination kinetics may in part be due to different underlying mechanisms. It has been shown that archetype I (ToxoDB#10) disseminates as ‘free tachyzoites’, while archetypes II (ToxoDB#1) and III (ToxoDB#2) are shuttled by leukocytes (Lambert and Barragan, 2010; Delgado Betancourt et al., 2019). Dissemination as ‘free tachyzoites’ may be facilitated by the high lytic capacity of the acutely virulent ToxoDB#10, which causes rapid egress of tachyzoites from infected host cells. Since it was shown that EQ40 and K1 do have a higher lytic capacity as compared to BGD18 (ToxoDB#1), G13 and EQ39 (ToxoDB#54) (Uzelac et al., 2020), albeit much lower than that of RH (ToxoDB#10), a greater number of EQ40 and K1 tachyzoites may disseminate as ‘free tachyzoites’. However, given the low dose inoculum and that the peritoneal cavity consists of B-1 cells, large peritoneal macrophages (LPM), small peritoneal macrophages (SPM), B-2 cells, T cells, NK cells, DCs and granulocytes (Cassado Ados et al., 2015), dissemination of all genotypes was likely facilitated primarily by immune cells. As it was previously shown that some 60% of infected cells post intraperitoneal inoculation of *T. gondii* tachyzoites are macrophages and given that macrophages make up nearly 30% of the cells in the peritoneal cavity, macrophages may have played a significant role in dissemination (Jensen et al., 2011; Cassado Ados et al., 2015). Another possibility which may explain the dissemination kinetics and cannot be ruled out is that virulent genotypes overcome the limits of detection by PCR and other methods more rapidly as compared to those of low virulence, due to different proliferation rates. It would thus appear that tachyzoites of more virulent genotypes are detected at earlier time points in different tissues as compared to tachyzoites of low virulence (Djurković-Djaković et al., 2012). Indeed, the lineage III genotypes examined here have vastly different proliferation rates (Uzelac et al., 2020) and it has been demonstrated previously that tachyzoites of virulent genotypes proliferate faster as compared to those of low virulence (Kaufman et al., 1959; Radke et al., 2001). In light of that, definitive conclusions regarding dissemination capacity need to be made with caution.

At 24 h p.i., differential induction of cytokine expression in the examined tissues (**Figure 1**) was concordant with the dissemination results (**Table 2**). Although the cytokine levels secreted by immune cells in the tested tissues were not evaluated, the expression data indicate that transcriptional activation of immune cells occurs rapidly p.i. To check which is the earliest time point p.i. for detection of T-bet, 24 h and three days p.i. were analyzed first. The last time point, 10 days p.i. was analyzed to ascertain the trend of intensity of the immune response. The highest levels of all three cytokines and T-bet were detected in the CLN of mice infected with the genotype with the highest CM, EQ40. The differences were not statistically significant, due to high variability of expression levels between individual mice, which is expected in outbred strains (**Figures 1**, **3**). The results indicate that immune cells are strongly activated rapidly after infection with this genotype. Surprisingly, the same is not evident in the mice infected with the other intermediately virulent genotype, K1, save for somewhat higher T-bet levels in comparison to those detected in the CLN of mice infected with the genotypes of low virulence. In fact, based on the levels of IFNγ and IL12-p40 expression elicited by K1, it appears that a stronger activation has occurred in the mice infected with BGD18 (ToxoDB#1) at 24 h post inoculation. Early secretion of pro- and anti-inflammatory cytokines *in vitro* (Saeij et al., 2007) and in mouse sera *in vivo* (Djurković-Djaković et al., 2006) has been reported before for ToxoDB#1 and may in part be mechanistically explained by the type II allele at the ROP16 and GRA15 loci (Rosowski et al., 2011). As both IFNγ and IL12-p40 are required early for initiating cellular immunity and controlling tachyzoite proliferation to ensure host survival, the immune response induced by BGD18 (ToxoDB#1) provides the host with an advantage over the parasite, which may also explain the low virulence in mice. The expression results suggest that EQ40, just like BGD18 (ToxoDB#1), elicits a host protective response early on, but of far greater intensity. As EQ40 (and all other genotypes analyzed in this study) carries a type I allele at both loci, yet induces significant expression of pro-inflammatory cytokines and IL-10, other mechanisms which regulate gene expression must be in place. Interestingly, the kinetics of expression of the three cytokines and T-bet in the CLN indicate that the intensity of the response to EQ40 wanes over time, while it becomes amplified for BGD18 (ToxoDB#1) and K1, the other intermediately virulent genotype of lower CM than EQ40. In fact, a spike in cytokine levels is evident between day seven and day ten in the CLN of the mice infected with BGD18 (ToxoDB#1) and K1. A trend is difficult to discern for G13 and EQ39 (ToxoDB#54) but with IFNγ down regulated until day seven and low expression of IL12-p40 throughout the time course with comparatively much higher levels of IL-10, the immune response is milder, which is consistent with the low virulence and low CM phenotype.

Relative cytokine expression was analyzed in infection (with tachyzoites of low virulence) as of the first time point, but as parasites are infrequently detected in the brain and spleen 24 h and three days p.i., induction of expression could not be detected in the majority of the mice at these time points (data not shown). Since the dissemination peak for tachyzoites of low virulence occurred seven days p.i., the cytokine expression profile at this time point is presented. The highest levels of IFNγ and IL12-p40 were detected in the brain homogenates of the mice infected with EQ40, while IL-10 was down-regulated in all infected mice. At the same time point, BAG1 transcripts could not be detected in any of the mice infected with EQ40, despite the presence of parasite gDNA in the brain, while interestingly, BAG1 was detected in the brain of 2/3 mice infected with BGD18 (ToxoDB#1) and 1/3 mice infected with EQ39 (ToxoDB#54) (**Table 3**). Although the presence of tissue cysts has not been confirmed visually in this study (and can hardly be expected to using conventional microscopy), detection of the BAG1 transcript indicates that stage conversion has happened and/or is underway. Interestingly, the presence of BAG1 best correlates with the absence and/or low levels of IFNγ expression. Recently it was shown that *in vitro* certain subsets of murine and human neurons can kill intracellular parasites in response to IFNγ stimulation prior to infection (Chandrasekaran et al., 2022). The implication of this finding *in vivo* may be that conversion and encystation must happen early after infection to avoid high IFNγ conditions which favor clearance. Thus genotypes which induce copious amounts of IFNγ in the brain early, such as EQ40, may undergo stage conversion and complete encystation at a later time point, once the level drops. Unfortunately, as BAG1 and even SAG1 transcripts could not be consistently detected at the investigated time points, delayed encystation by EQ40 could not be conclusively ascertained. As in the brain, the highest level of IFNγ was detected in the spleens of mice infected with EQ40, while IL12-p40 was down-regulated. Indeed, IL12-p40 was only up-regulated in the spleens of the mice infected with BGD18 (ToxoDB#1), while IL-10 was up-regulated in the spleens of mice infected with BGD18 (ToxoDB#1) and G13 (**Figure 2**).

The combined gene expression results from the CLN, brain and spleen indicate that infection by EQ40 tachyzoites is characterized by high IFNγ expression, which together with high T-bet expression, points to a strong systemic inflammatory reaction, possibly resulting in immunopathology (Yap and Sher, 1999). Unlike in the CLN, IL-10 is down-regulated in both brain and spleen of the mice 7 days post infection, suggesting a possible absence of a balancing regulatory response in these tissues and increasing the likelihood for the development of a certain degree of pathology. Disease manifestations such as colitis and/or encephalitis have been observed even after infection with ToxoDB#1 in different mouse strains (Liesenfeld, 2002; Djurković-Djaković et al., 2006), suggesting that even genotypes of low virulence and low CM can cause immunopathology. As histopathology was not performed in this study, it is unclear whether even a low dose infection with EQ40 results in tissue damage. However, as previous experiments showed that all mice survive well into chronic infection when infected with 10 tachyzoites, even if there is any tissue damage, this will not be devastating for the host (Uzelac, 2021 dissertation). Interestingly, in both the brain and spleens of the mice infected with K1, the other intermediately virulent genotype, the expression of all three cytokines was down regulated, suggesting that immunopathology does not contribute to its virulence. It was shown previously that when using higher doses of tachyzoites for infection, 76.1% of the mice infected with K1 die up to day 15, as compared to 88% with EQ40 (Uzelac et al., 2020). The low level of IFNγ seen early in infection with K1 may promote early conversion and encystation in the brain, but it is unclear whether the low levels observed prior to day 10 in the CLN and on day 7 in the spleen are sufficient to establish control over proliferation in these tissues, thus perhaps tipping the balance in favor of the parasite. The cytokine expression spike evident in the CLN of the mice infected with K1 at day ten, thus acts like an immunity boost to offset the imbalance and rescue the host. As there is no spike in cytokine expression evident in the mice infected with EQ40, the conditions likely favor tissue recovery from inflammation but may promote tachyzoite proliferation. The non-clonal lineage III genotypes used in this study have the same allele profile at some of the key virulence loci, but induce different immune responses, which are not in clear correlation with the virulence phenotype. The presented results suggest that some genotypes of *T. gondii* have the capacity to strongly stimulate the cellular immune response almost immediately after infection, while others do so gradually. As 4/5 genotypes investigated here exhibit a gradually amplifying cellular immune response, it stands to reason that this is a common trend, while an intense early response seems to be characteristic only for genotypes of high CM, like EQ40. To conclude, this study showed that there is a high diversity among lineage III genotypes in terms of virulence phenotype, CM and induced immune response.

## Data availability statement

The raw data supporting the conclusions of this article will be made available by the authors, without undue reservation.

## Ethics statement

The animal study was approved by Veterinary Directorate, Ministry of Agriculture, Forestry and Water Economy of Serbia. The study was conducted in accordance with the local legislation and institutional requirements.

## Author contributions

AU: Conceptualization, Formal analysis, Investigation, Methodology, Validation, Visualization, Writing – original draft. IK: Data curation, Methodology, Supervision, Writing – review & editing. OD-D: Conceptualization, Funding acquisition, Project administration, Resources, Supervision, Writing – review & editing.
